# The worldwide clinical trial research response to the COVID-19 pandemic - the first 100 days

**DOI:** 10.12688/f1000research.26707.2

**Published:** 2020-10-26

**Authors:** Perrine Janiaud, Cathrine Axfors, Janneke van't Hooft, Ramon Saccilotto, Arnav Agarwal, Christian Appenzeller-Herzog, Despina G. Contopoulos-Ioannidis, Valentin Danchev, Ulrich Dirnagl, Hannah Ewald, Gerald Gartlehner, Steven N. Goodman, Noah A. Haber, Angeliki Diotima Ioannidis, John P. A. Ioannidis, Mark P. Lythgoe, Wenyan Ma, Malcolm Macleod, Mario Malički, Joerg J. Meerpohl, Yan Min, David Moher, Blin Nagavci, Florian Naudet, Christiane Pauli-Magnus, Jack W. O'Sullivan, Nico Riedel, Jan A. Roth, Mandy Sauermann, Stefan Schandelmaier, Andreas M. Schmitt, Benjamin Speich, Paula R. Williamson, Lars G. Hemkens

**Affiliations:** 1 Meta-Research Innovation Center at Stanford (METRICS), Stanford University,, Stanford, California, USA; 2 Department of Clinical Research, University of Basel, Basel, Switzerland; 3 Department for Women’s and Children’s Health, Uppsala University, Uppsala, Sweden; 4 Amsterdam University Medical Center, Amsterdam University, Amsterdam, The Netherlands; 5 Department of Medicine, University of Toronto, Toronto, Ontario, Canada; 6 University Medical Library, University of Basel, Basel, Switzerland; 7 Division of Infectious Diseases, Department of Pediatrics, Stanford University School of Medicine, Stanford, California, USA; 8 Stanford Prevention Research Center, Department of Medicine,, Stanford University School of Medicine, Stanford, California, USA; 9 QUEST Center for Transforming Biomedical Research, Berlin Institute of Health, Berlin, Germany; 10 Department for Evidence-based Medicine and Evaluation, Danube University Krems, Krems, Austria; 11 RTI International, Research Triangle Park Laboratories, Raleigh, North Carolina, USA; 12 Stanford University School of Medicine, Stanford University School of Medicine, Stanford, California, USA; 13 Department of Epidemiology and Population Health, Stanford University School of Medicine, Stanford, California, USA; 14 Molecular Toxicology Interdepartmental Program, University of California, Los Angeles, Los Angeles, California, USA; 15 Meta-Research Innovation Center Berlin (METRIC-B), Berlin Institute of Health, Berlin, Germany; 16 Department of Surgery & Cancer, Imperial College London, London, UK; 17 Centre for Clinical Brain Sciences, University of Edinburgh, Edinburgh, UK; 18 Institute for Evidence in Medicine, Medical Center and Faculty of Medicine, University of Freiburg, Freiburg, Germany; 19 Cochrane Germany, Cochrane Germany Foundation, Freiburg, Germany; 20 Centre for Journalology, Clinical Epidemiology Program, Ottawa Health Research Institute, Ottawa, Canada; 21 CHU Rennes, Inserm, CIC 1414 [(Centre d’Investigation Clinique de Rennes)],, University of Rennes 1, Rennes, France; 22 Division of Cardiology, Department of Medicine, Stanford University School of Medicine, Stanford, California, USA; 23 Division of Infectious Diseases and Hospital Epidemiology, University Hospital Basel, Basel, Switzerland; 24 Department of Health Research Methods, Evidence, and Impact, McMaster University, Hamilton, Ontario, Canada; 25 Deparment of Medical Oncology, University Hospital Basel, Basel, Switzerland; 26 Centre for Statistics in Medicine, Nuffield Department of Orthopaedics, Rheumatology and Musculoskeletal Sciences, University of Oxford, Oxford, UK; 27 MRC/NIHR Trials Methodology Research Partnership, University of Liverpool, Liverpool, UK

**Keywords:** COVID-19, clinical research agenda, hydroxychloroquine

## Abstract

**Background**: Never before have clinical trials drawn as much public attention as those testing interventions for COVID-19. We aimed to describe the worldwide COVID-19 clinical research response and its evolution over the first 100 days of the pandemic.

**Methods: **Descriptive analysis of planned, ongoing or completed trials by April 9, 2020 testing any intervention to treat or prevent COVID-19, systematically identified in trial registries, preprint servers, and literature databases. A survey was conducted of all trials to assess their recruitment status up to July 6, 2020.

**Results:** Most of the 689 trials (overall target sample size 396,366) were small (median sample size 120; interquartile range [IQR] 60-300) but randomized (75.8%; n=522) and were often conducted in China (51.1%; n=352) or the USA (11%; n=76). 525 trials (76.2%) planned to include 155,571 hospitalized patients, and 25 (3.6%) planned to include 96,821 health-care workers. Treatments were evaluated in 607 trials (88.1%), frequently antivirals (n=144) or antimalarials (n=112); 78 trials (11.3%) focused on prevention, including 14 vaccine trials. No trial investigated social distancing. Interventions tested in 11 trials with >5,000 participants were also tested in 169 smaller trials (median sample size 273; IQR 90-700). Hydroxychloroquine alone was investigated in 110 trials. While 414 trials (60.0%) expected completion in 2020, only 35 trials (4.1%; 3,071 participants) were completed by July 6. Of 112 trials with detailed recruitment information, 55 had recruited <20% of the targeted sample; 27 between 20-50%; and 30 over 50% (median 14.8% [IQR 2.0-62.0%]).

**Conclusions:** The size and speed of the COVID-19 clinical trials agenda is unprecedented. However, most trials were small investigating a small fraction of treatment options. The feasibility of this research agenda is questionable, and many trials may end in futility, wasting research resources. Much better coordination is needed to respond to global health threats.

## Introduction

On December 31, 2019, the World Health Organization (WHO) China Country Office was informed of pneumonia cases of unknown etiology ^1^; on January 30, 2020, the WHO declared coronavirus disease 2019 (COVID-19) ^2^ a public health emergency and on March 11 a pandemic ^3^. Radical public health measures, including quarantine, social distancing, school and workplace closures, and others have been implemented worldwide, affecting the lives of billions of people. The pandemic resulted in rapid generation and dissemination of studies and their results ^4^. However, information on trials that are planned, ongoing, finished, or published are spread across trial registries, preprint servers, publication databases and other repositories. In June, 2020, the number of ongoing trials outweighed by far completed trials; however, no overview of COVID-19 trials has followed up on actual enrolment in ongoing trials ^5– 8^.

We established the COVID-evidence platform ( www.covid-evidence.org) to collect this information in a central database of COVID-19 trials testing any interventions for treatment or prevention. We used COVID-evidence to describe the worldwide clinical research response to COVID-19, its evolution over the first 100 days since the first cases were officially reported, and the expected feasibility and risk of waste of resources. We describe the trials’ characteristics, their place in the research landscape, and how they changed over time.

## Methods

### Data sources

COVID-evidence includes trials from international registries (ClinicalTrials.gov, WHO International Clinical Trials Registry Platform [ICTRP]), preprint servers (medRxiv, bioRxiv), PubMed, the WHO COVID-19 literature database, and a listing of all trials with ethical approval in Switzerland ^9^.

### Identification and selection of trials

Our protocol and details on the search strategies and specific definitions used for COVID-evidence are available on the Open Science Framework (OSF) ^10^. We searched the relevant data sources using peer-reviewed search strategies developed by information specialists. Results from literature databases and preprint servers were pre-screened by a single reviewer who excluded unsuitable publications (e.g. opinion papers or observational, non-interventional studies). Cases with unclear eligibility were discussed by at least three reviewers until consensus was reached.

We included any planned, ongoing or completed trial that tested any intervention to treat or prevent COVID-19 in humans that was registered or published within the first 100 days of the COVID-19 outbreak, i.e. after the first cases reported to the WHO to 100 days later (January 1 to April 9, 2020). We considered as a trial any study prospectively assigning an intervention ^11^. This included randomized and non-randomized, controlled or non-controlled trials regardless of language, geographical region, or setting. Epidemiological studies or studies of diagnostic test accuracy (without any health-related outcome) were excluded.

### Data extraction

For each trial, we extracted dates of registration and publication, design characteristics and details of the population, intervention, comparison, outcomes, geographic region, funding and setting. We categorized drugs and biologicals according to major pharmacological classes and main clinical indications.

A team of 19 reviewers (including clinicians, clinical researchers, clinical pharmacologists, meta-researchers and systematic reviewers) either manually extracted information or verified information that was obtained with automatic data scraping methods. All details on scraping, variable definitions and extraction/verification procedures are available on OSF ^10, 12^. For all trials registered up to April 9, 2020, we extracted data through April 30. The status for each trial was updated on July 6, 2020 (using ClinicalTrials.gov where possible; if not, we used the ICTRP; for trials originally registered in the Chinese Clinical Trial Registry available through ICTRP we used the former if it was more up to date).

When a trial had entries in different data sources, we gave first priority to publications, second to preprints, and third to registries (here, ClinicalTrials.gov was preferred).

### Author requests on enrolment

From May 12 to July 3, 2020, we emailed the corresponding investigators of all trials, except discontinued ones, inquiring about their enrolment accrual. Replies were collected up to July 6, 2020.

### Statistical analyses

All analyses were descriptive and reported as percentages, medians (interquartile range, IQR) or means. We used Ninox (Ninox Software GmbH, Berlin, Germany; version 2.6), and R (version 3.6).

## Results

We identified 683 trials registered or published over the pandemic’s first 100 days, testing interventions to treat or prevent COVID-19 (see *Underlying data*) ^12^ with a total planned sample size of 394,146 participants. As of July 6, 2020, 19 trials (including 4,378 participants) had been completed and had published results, and 16 were completed without available results (5,173 participants). Twenty-nine (4.2%) were active but no longer recruiting (58,589 participants), 381 (55.8%) started recruiting (215,807 participants), 174 (25.5%) had not yet started (97,406 participants), 50 (7.3%) were discontinued (12,048 participants), and 4 (0.6%) were terminated (577 participants). The status was unknown for 10 (1.5%; 168 participants).

### General characteristics

The 683 trials’ median target sample size was 118 (IQR 60 to 300; Table 1); 40.7% (n=280) planned to enroll fewer than 100 participants, 8.2% (n=56) over 1,000, and 1.6% (n=11) over 5,000 (see *Underlying data*) ^12^. 75.5% (n=516) trials were randomized and 59.4% (n=406) did not use blinding ( Table 1). Randomized trials were on average almost three times larger than non-randomized trials (median sample size 144 vs. 50).

**Table 1.** Trial characteristics: total and stratified by purpose of trial, recruiting status and top 5 countries in registration numbers.

Trial characteristics	Total (n=683)	For treatment ^a^ (n=602)	For prevention ^a^ (n=77)	Not yet recruiting (n=174)	Recruiting (n=381)	China (n=351)	United States (n= 76)	France (n=34)	Spain (n=21)	International (n=21)
**Randomized ^b^**	516 (75.5%)	457 (75.9%)	55 (71.4%)	132 (75.9%)	294 (77.2%)	257 (73.2%)	53 (69.7%)	29 (85.3%)	21 (100%)	20 (95.2%)
Two arms	395 (57.8%)	356 (59.1%)	35 (45.4%)	101 (58%)	226 (59.3%)	205 (58.4%)	40 (52.6%)	21 (61.8%)	17 (80.9%)	10 (47.6%)
Three or more arms	117 (17.1%)	98 (16.3%)	19 (24.7%)	31 (17.8%)	66 (17.3%)	52 (14.8%)	13 (17.1%)	8 (23.5%)	4 (19%)	9 (42.9%)
Number of arms not reported	4 (0.6%)	3 (0.5%)	1(1.3%)	0 (0%)	2 (0.5%)	0 (0%)	0 (0%)	0 (0%)	0 (0%)	1 (4.8%)
**Non-randomized**	161 (23.6%)	139 (23.1%)	22 (28.6%)	41 (23.6%)	87 (22.8%)	91 (25.9%)	23 (30.3%)	5 (14.7%)	0 (0%)	1 (4.8%)
Single arm	101 (14.8%)	88 (14.6%)	13 (16.8%)	25 (14.4%)	54 (14.2%)	49 (13.9%)	18 (23.7%)	4 (11.8%)	0 (0%)	1 (4.8%)
Two arms	47 (6.9%)	40 (6.6%)	7 (9.1%)	15 (8.6%)	23 (6%)	34 (9.7%)	3 (3.9%)	0 (0%)	0 (0%)	0 (0%)
Three or more arms	13 (1.9%)	11 (1.8%)	2 (2.6%)	1 (0.6%)	10 (2.6%)	8 (2.3%)	2 (2.6%)	1 (2.9%)	0 (0%)	0 (0%)
**Blinding**										
None	406 (59.4%)	366 (60.8%)	38 (49.4%)	99 (56.9%)	219 (57.5%)	208 (59.3%)	41 (53.9%)	23 (67.6%)	11 (52.4%)	10 (47.6%)
Double blind	134 (19.6%)	110 (18.3	24 (31.2%)	24 (13.8%)	82 (21.5%)	34 (9.7%)	29 (38.2%)	9 (26.5%)	8 (38.1%)	10 (47.6%)
Single blind	36 (5.3%)	29 (4.8%)	7 (9.1%)	9 (5.2%)	25 (6.6%)	11 (3.1%)	5 (6.6%)	2 (5.9%)	1 (4.8%)	1 (4.8%)
Outcome only	8 (1.2%)	3 (0.5%)	5 (6.5%)	5 (2.9%)	2 (0.5%)	3 (0.9%)	1 (1.3%)	0 (0%)	1 (4.8%)	0 (0%)
Not reported	99 (14.5%)	94 (15.6%)	3 (3.9%)	37 (21.3%)	53 (13.9%)	95 (27.1%)	0 (0%)	0 (0%)	0 (0%)	0 (0%)
**Planned sample size** ^c^										
Total	394,146	186,189	204,641	97,406	215,807	70,285	49,855	13,276	13,005	138,120
Median [IQR]	118 [60-300]	100 [50-240]	440 [142.5-1,205]	100 [60-200]	122 [60-350]	90 [40-160]	160 [50-500]	239.5 [100-553]	200 [120-440]	1,200 [240-4,500]
Min-max	4-55,000	4-12,000	4-55,000	10-55,000	5-20,000	4-20,000	5-15,000	11-1,300	24-4,000	20-55,000
**Intervention**										
Drug	385 (56.4%)	345 (57.3%)	38 (49.4%)	76 (43.7%)	231 (60.6%)	138 (39.3%	55 (72.4%)	28 (82.4%)	19 (90.5%)	20 (95.2%)
Traditional medicine	108 (15.8%)	100 (16.6%)	8 (10.4%)	41 (23.6%)	57 (15%)	101 (28.8%	0 (0%)	0 (0%)	0 (0%)	0 (0%)
Biological	79 (11.6%)	77 (12.8%)	1 (1.3%)	19 (10.9%)	40 (10.5%)	50 (14.2%)	9 (11.8%)	1 (2.9%)	1 (4.8%)	0 (0%)
Procedure	31 (4.5%)	30 (5%)	1 (1.3%)	12 (6.9%)	15 (3.9%)	15 (4.3%)	3 (3.9%)	4 (11.8%)	0 (0%)	0 (0%)
Device	11 (1.6%)	8 (1.3%)	2 (2.6%)	3 (1.7%)	6 (1.6%)	2 (0.6%)	2 (2.6%)	0 (0%)	1 (4.8%)	0 (0%)
Vaccine	13 (1.9%)	0 (0%)	13 (16.9%)	2 (1.1%)	8 (2.1%)	6 (1.7%)	2 (2.6%)	0 (0%)	0 (0%)	0 (0%)
Multiple intervention ^d^	18 (2.5%)‡	15(2.5%)	3 (3.9%)	5 (3%)	11(2.9%)	13 (3.8%)	1 (1.3%)	0 (0%)	0 (0%)	1 (4.8%)
Other	35 (5.1%)	25 (4.2%)	10 (13%)	15 (8.6%)	13 (3.4%)	24 (6.8%)	4 (5.3%)	1 (2.9%)	0 (0%)	0 (0%)
Unclear	3 (0.4%)	2 (0.3%)	1 (1.3%)	0 (0%)	0 (0%)	2 (0.6%)	0 (0%)	0 (0%)	0 (0%)	0 (0%)
**Control**										
Standard of care / No intervention	310 (45.4%)	284 (47.2%)	23 (29.9%)	92 (52.9%)	175 (45.9%)	187 (53.3%)	19 (25%)	15 (44.1%)	7 (33.3%)	6 (28.6%)
Placebo	130 (19%)	103 (17.1%)	27 (35.1%)	30 (17.2%)	73 (19.2%)	46 (13.1%)	24 (31.6%)	7 (20.6%)	5 (23.8%)	11 (52.4%)
Other active intervention	118 (17.3%)	108 (17.9%)	9 (11.7%)	25 (14.4%)	70 (18.2%)	62 (17.7%)	12 (15.8%)	7 (20.6%)	6 (27.3%)	2 (9.5%)
No control (single arms)	101 (14.8%)	88 (14.6%)	13 (16.9%)	25 (14.4%)	54 (14.2%)	49 (14%)	18 (23.7%)	4 (11.8%)	0 (0%)	1 (4.8%)
Other and combination ^e^	12 (1.8%)	9 (1.5%)	3 (3.9%)	2 (1.1%)	10 (2.6%)	2 (0.6%)	3 (3.9%)	1 (2.9%)	2 (9.5%)	1 (4.8%)
Unclear	12 (1.8%)	10 (1.7%)	2 (2.6%)	0 (0%)	2 (0.5%)	5 (1.4%)	0 (0%)	0 (0%)	1 (4.8%)	1 (0.3%)
**Mortality outcome**										
Primary outcome	98 (14.3%)	95 (15.8%)	3 (3.9%)	21 (12.1%)	63 (16.5%)	34 (9.7%)	3 (3.9%)	16 (47.1%)	6 (28.6%)	5 (23.8%)
Secondary outcome	220 (32.2%)	208 (34.6%)	10 (13%)	43 (24.7%)	129 (33.9%)	79 (22.5%)	31 (40.8%)	12 (35.3%)	9 (42.9%)	10 (47.6%)
Not reported as outcome	365 (53.4%)	299 (49.7%)	64 (83.1%)	110 (63.2%)	189 (49.6%)	238 (67.8%)	42 (55.3%)	6 (17.6%)	6 (28.6%)	6 (28.6%)
**Participants**										
Inpatient	521 (76.3%)	502 (83.4%)	17 (22.1%)	129 (74.1%)	293 (76.9%)	275 (78.3%)	46 (60.5%)	30 (88.2%)	14 (66.7%)	17 (81%)
Outpatient	55 (8.1%)	37 (6.1%)	18 (23.4%)	19 (10.9%)	28 (7.3%)	25 (7.1%)	10 (13.2%)	1 (2.9%)	1 (4.8%)	2 (9.5%)
Healthcare workers	24 (3.5%)	0 (0%)	24 (31.2%)	5 (2.9%)	14 (3.7%)	3 (0.9%)	7 (9.2%)	2 (5.9%)	4 (19%)	1 (4.8%)
Healthy participants	9 (1.3%)	1 (0.2%)	8 (10.4%)	3 (1.7%)	5 (11.3%)	3 (0.9%)	3 (3.9%)	0 (0%)	1 (4.8%)	0 (0%)
Multiple/mixed	15 (2.2%)	8 (1.3%)	5 (6.5%)	4 (2.3%)	9 (2.4%)	7 (2%)	4 (5.3%)	0 (0%)	1 (4.8%)	0 (0%)
Unclear	59 (8.6%)	54 (9%)	5 (6.5%)	14 (8%)	32 (8.4%)	38 (10.8%)	6 (7.9%)	1 (2.9%)	0 (0%)	1 (4.8%)
**Funding type**										
Public/not-for-profit	263 (38.5%)	241 (40%)	21 (27.3%)	76 (43.7%)	157 (41.2%)	178 (50.7%)	9 (11.8%)	13 (38.2%)	8 (38.1%)	5 (23.8%)
Industry/for-profit	66 (9.7%)	61 (10.1%)	5 (6.5%)	17 (9.8%)	38 (10%)	29 (8.3%)	9 (11.8%)	1 (2.9%)	1 (4.8%)	6 (28.6%)
Both	11 (1.6%)	11 (1.8%)	0 (0%)	2 (1.1%)	7 (1.8%)	8 (2.3%)	0 (0%)	1 (2.9%)	0 (0%)	0 (0%)
Reported but unclear ^f^	286 (41.9%)	243 (40.4%)	41 (53.2%)	62 (35.6%)	156 (40.9%)	104 (29.6%)	51 (67.1%)	16 (47.1%)	10 (47.6%)	9 (42.9%)
Not reported	57 (8.3%)	46 (7.6%)	10 (13%)	17 (9.8%)	23 (6%)	32 (9.1%)	7 (9.2%)	3 (8.8%)	2 (9.5%)	1 (4.8%)

Additional categories such as other countries can be found in the extended data ^a^ 4 trials assessed interventions for both treatment and intervention ^b^ For 6 trials the randomization was unclear ^c^ The sample size is missing for 15 trials ^d^ Some trials compared different types of interventions: 3 trials biologicals and drugs; 6 trials drugs and traditional medicine; 5 trials other interventions and drugs; 1 trial other interventions and traditional medicine; 2 trials procedures and drugs; 1 trial vaccine and device ^e^ Included for example use trials that used a standard of care arm and an active control arm ^f ^a public/not-for-profit sponsor was reported but the absence of an industrial sponsor does not exclude an industrial funder

Although few trials focused on health-care workers (3.5% [n=24]), they were larger: 95,621 planned health-care workers (median 690 [IQR 390 to 2,600]) versus 155,221 planned patients (median 100 [IQR 50 to 240]) for the inpatient trials (76.3% [n=521]) ( Table 1). Overall, 46.5% of the trials intended to use mortality as a primary (n=98) or secondary outcome (n=220; Table 1), and 53•4% (n=365) did not specify mortality as an outcome. Out of the 521 inpatient trials, 55.7% (n=290) planned on reporting mortality as an outcome.

### Interventions to treat COVID-19

**Figure 1.**
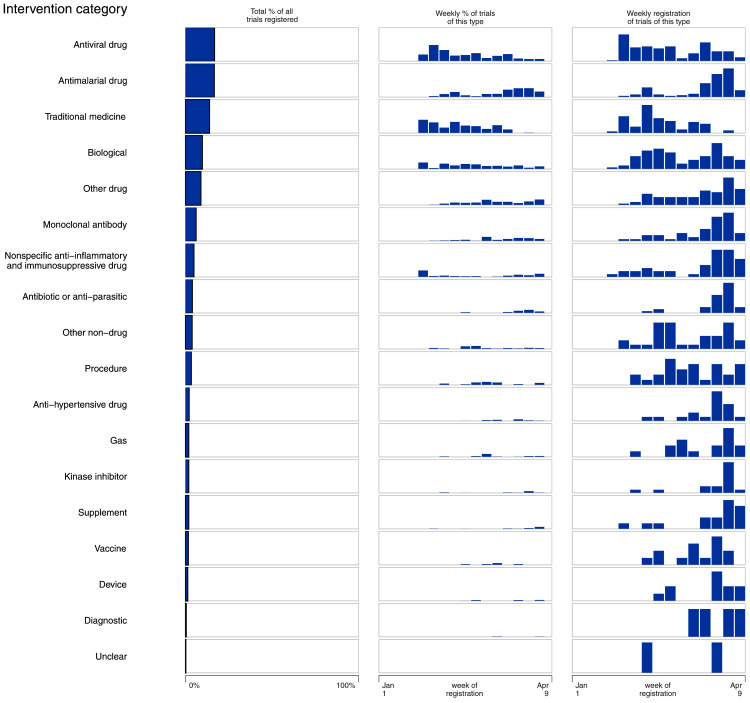
Number of trials assessing the different intervention categories. Interventions for treatment assessed in more than 25 trial: antiviral drugs were assessed in 141 trials; (e.g. lopinavir/ritonavir [n=45]), antimalarial drugs in 111 trials; (e.g. hydroxychloroquine [n=84]), monoclonal antibodies in 51 trials (e.g. tocilizumab [n=25]), traditional medicine in 106 trials, other drug intervention in 70 trials, nonspecific anti-inflammatory/immunosuppressive drugs in 42 trials (e.g. colchicine [n=4]), antibiotic/anti-parasitic drugs in 34 trials (e.g. azithromycin [n=28]), biologicals in 80 trials (e.g. convalescent plasma [n=27]), procedures in 28 trials (e.g. renal replacement therapy [n=4]), other non-drug interventions in 267 trials (e.g. physical activity). More information can be found in the Extended data ^12^. The first column represents the proportion of all trials that were of the specified type. The second column represents the proportion of all trials registered that week that were of the specified type (i.e. within week, between trial types). The third column represents the distribution of when trials of this type were registered (i.e. within trial type, between week), and can be interpreted as either a percentage or count (not specified). A trial might assess more than one intervention category and detail for prevention and treatment trials are given in the extended data ^12^.

Out of the 683 trials, 602 (88.1%) assessed treatment interventions (186,189 planned patients); drugs were more frequent (345 trials [57.3%]), encompassing a vast range of substances. The two most common pharmacological classes were antiviral drugs (assessed in 141 trials; e.g. lopinavir/ritonavir [n=45]) and antimalarial drugs (111 trials; e.g. hydroxychloroquine [n=84]). There were 106 trials investigating traditional medicine and 70 exploring highly diverse pharmaceuticals of various classes, e.g. bismuth potassium citrate, ebastine, pirfenidone, dipyridamole and hydrogen peroxidase. Regarding non-drug interventions, 28 investigated procedures (e.g. renal replacement therapy), eight devices (e.g. various respiratory devices), and 26 trials investigated other non-drug interventions (e.g. physical activity and pulmonary rehabilitation). ( Figure 1 and see *Extended data*) ^12^. The comparators were predominantly standard of care or no intervention (47.2% [n=284]), placebo (17.1% [n=103]) or other interventions (17.9%; [n=108]) ( Table 1).

### Interventions to prevent COVID-19

Overall, 77 trials (11.3%) focused on prevention (204,641 planned participants), mainly prophylactic drug use (n=41), vaccines (n=14; 9 already started recruitment; see *Extended data*) ^12^ and non-pharmaceutical interventions (n=10) (e.g. masks or the use of media and influencers in people’s compliance to hygienic practices). Four trials (0.6%) assessed interventions both for prevention and for treatment. No trial planned to assess benefits or harms of implementing or de-implementing any social distancing or lockdown measures.

### Time trends and global shift

The number of trials increased rapidly; on average 0.5 trials per day were registered in January, 8.1 in February, 8.3 in March, and 17.2 in April 2020.

**Figure 2.**
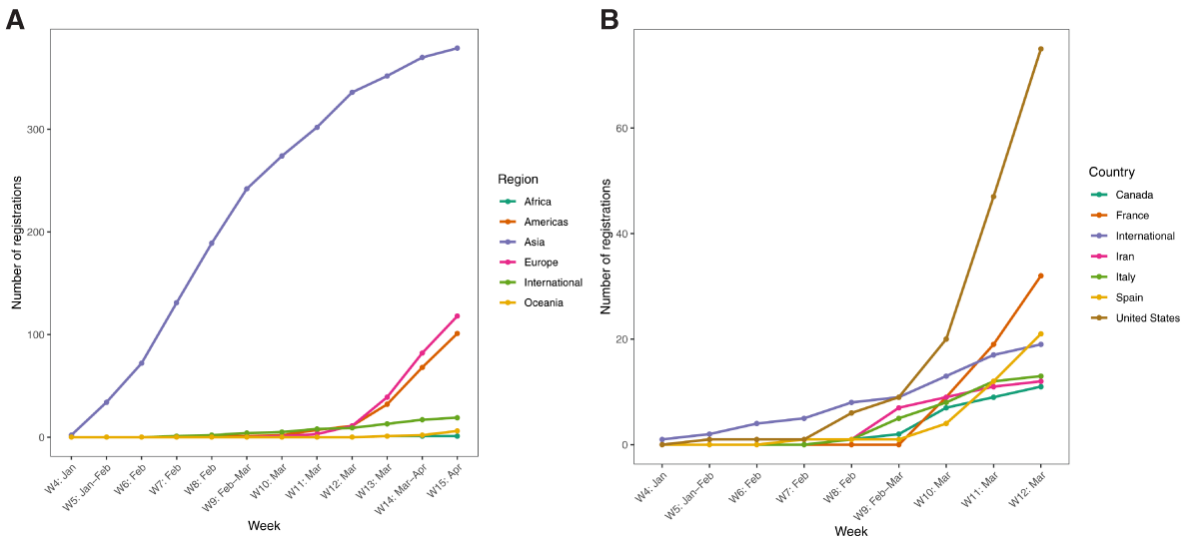
Cumulative number of registered trials over time ( **a**) by continent, and ( **b**) for countries with at least 10 registrations (excluding China). Four trials not shown were registered in 2019 or earlier, with a study design subsequently adapted to address COVID-19 (EUCTR2015-002340-14-NL; NCT03680274; NCT03331445; and NCT03808922). For 18 trials the registration date was unknown.

Trials were conducted in 41 countries and through international collaborations ( Table 1; see *Extended data*) ^12^. Half were from China (51.4% [n=351]), which dominated initially ( Figure 2); starting March 2020, more trials came from other countries. Trial characteristics were similar across the five most frequent geographical locations (China, USA, France, Spain and international) contributing to 73.6% (n=503) of the global trial research ( Table 1). Traditional medicine was assessed in 30.5% of trials from China (n=107) but rarely in other countries.

**Figure 3.**
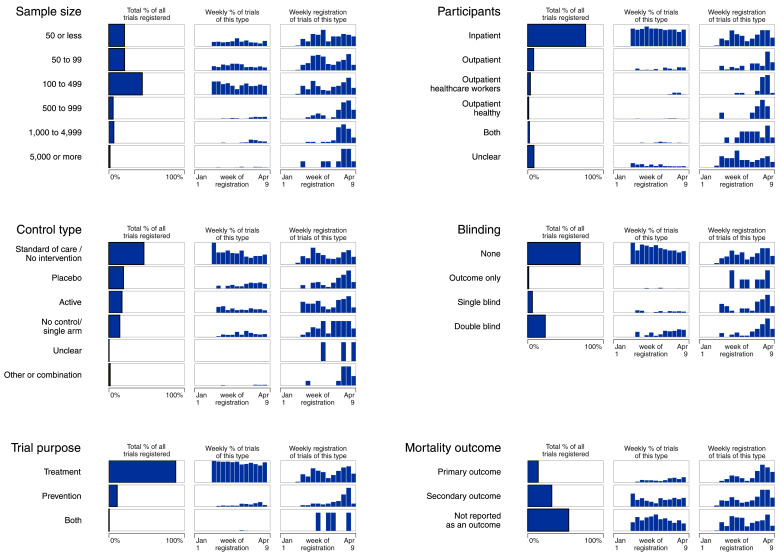
Proportion of trials stratified according to ( **a**) sample size, ( **b**) type of participants, ( **c**) type of control, ( **d**) type of blinding, ( **e**) purpose of the trial, and ( **f**) use of mortality as outcome. The first column represents the proportion of all trials that were of the specified type. The second column represents the proportion of all trials registered that week that were of the specified type (i.e. within week, between trial types). The third column represents the distribution of when trials of this type were registered (i.e. within trial type, between week), and can be interpreted as either a percentage or count (not specified).

Larger trials were initiated later. In February, 5.1% of trials included more than 500 participants in contrast to 18.6% of trials in March ( Figure 3). Later trials more often used blinding, placebo and mortality as primary outcome ( Figure 3). Participations of healthcare workers and healthy people also started later. When the proportion of trials from China decreased, so did trials assessing traditional medicine (from 46.9% to 0.9%) while the proportion of trials assessing drugs rose (from 40.6% to 77.7%). Antivirals came under investigation earlier than antimalarials ( Figure 1).

### Large trials

Out of the 683 trials, 6.6% (n=45) planned to enroll 1,000 to 5,000 participants. Most were randomized (88.9% [n=40]), assessed drugs (80%; n=36), and many were not blinded (53.3% [n=24]). Five were cluster-randomized. The top three regions were the United States (22.2%; n=10), France (11.1% [n=5]) and international collaborations (11.1% [n=5]) (see *Extended data*) ^12^.

Eleven (1.6%) trials, registered between February and April 2020 (seven for treatment and four for prevention), planned to enroll over 5,000 participants (see *Extended data*) ^12^. There were 10 randomized (one cluster RCT), eight not blinded and five conducted in multiple countries. These trials tested drugs (n=9), masks (n=1) and traditional medicine (n=1). Three trials are described as platform trials (i.e. WHO Solidarity trial ^13^, RECOVERY trial ^14^ and CROWN-CORONATION trial ^15^) and use an adaptive design.

**Figure 4.**
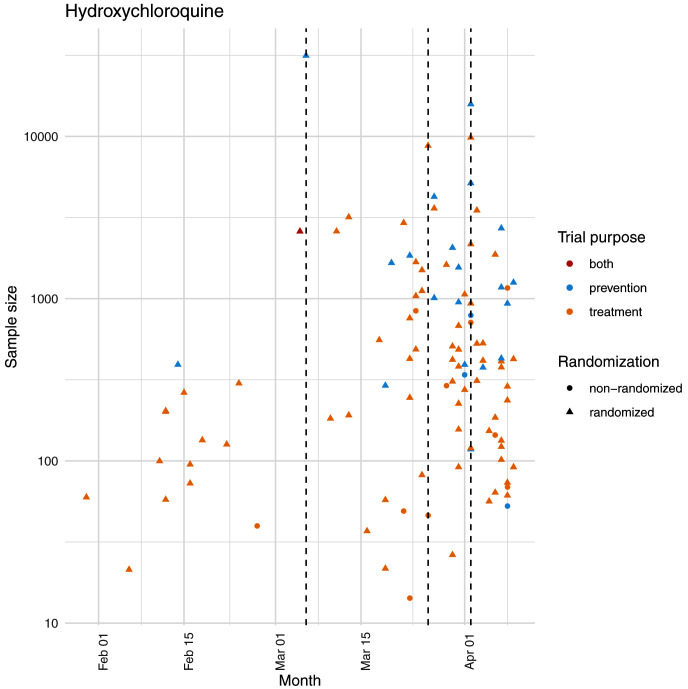
The 110 Trials assessing hydroxychloroquine for COVID-19 registered in the first 100 days of the pandemic. The dashed lines represent the registration of the four trials planning to enroll over 5,000 participants; two were registered on April 2, 2020. Out of the four trials planning on enrolling over 5,000 participants, two assessed hydroxychloroquine for treatment and two for prevention. Out of the 104 smaller trials, 82 assessed hydroxychloroquine for treatment, 21 for prevention and one for both treatment and prevention

Five drug interventions tested in these 11 larger trials were simultaneously investigated in over 20 smaller trials (see *Extended data*) ^12^. Overall, 167 trials (141 for treatment, 24 for prevention and two for treatment and prevention) with fewer than 5,000 participants assessed at least one intervention that was also assessed in a larger trial (median sample size 223 [IQR 80 to 540]; 132 had fewer than 1000 participants). For 103 of those (61.7%) the larger trial was registered before. For example, 104 trials with fewer than 5,000 participants tested hydroxychloroquine and 86 of them (82.7%) were registered after the first large trial testing this drug and 82 (78.8%) assessed hydroxychloroquine as treatment ( Figure 4; see *Extended data*) ^12^. These 104 trials had a median sample size of 334, but cumulatively, they planned to enroll as many patients as the four larger trials testing hydroxychloroquine (75,217 vs 77,000).

### Outlook

By the end of 2020, 413 trials (60.5%) with a total of 159,957 planned participants were expected to be completed (i.e. last patient, last visit), including 232 drug trials (97,282 participants) and 22 over-1,000 participants trials and five over-5,000 participants trials. For vaccines, five trials expected completion in 2020, five in 2021 and another four between 2022 and 2024 (see *Extended data*) ^12^. However, of the 270 trials that planned to start recruiting by the end of February, 190 started (70.4%), but 80 had not (as of July 6, 2020) (see *Extended data*) ^12^.

By July 6, 2020, we received enrolment information for 112 out of the 604 trials listed as planned or ongoing (18.7%). Of the 112 trials, 16 had not started recruiting although their start dates were overdue; one was discontinued. Among the 112 trials, 55 had recruited fewer than 20% of the target sample size, 27 between 20-50%, and 30 more than 50% (median recruitment 14.8% [IQR 2.0 to 62.0%]; median duration of recruitment 72 days [IQR 53.5 to 83 days])). Median recruitment was similar in treatment and prevention trials (15.9% (IQR 2 to 61.1%] vs 14.8% [IQR 4.3 to 62.5%]). For 19 trials, investigators mentioned difficulties in recruitment due to a fortunate decrease in the number of COVID-19 cases.

## Discussion

The global clinical research community has mounted a massive, unprecedented volume of research in response to the COVID-19 pandemic. Almost 700 trials within 100 days planned to include almost 400,000 participants globally. Many treatments were planned for investigation, mostly drugs, often antivirals, and sometimes substances that may seem rather unexpected for an infectious disease (e.g. colchicine or dipyridamole) reflecting the huge heterogeneity of disease manifestations and therapeutic targets ^16^. Few trials focused on prevention, but some were very large and focused on healthcare workers (i.e. 24.3% of planned trial participants are healthcare workers). Trials from China dominated the research agenda before research activities followed the spread of COVID-19 throughout the world. Most trials were planned as randomized, clearly demonstrating that such designs are possible within a pandemic ^17^ and within a very short time, unlike the 2014–2015 Ebola outbreak where only a few therapeutic trials had a randomized design, and none started within 100 days ^17^.

The emergence of 683 trials in a 100-day period is unparalleled. Between 250 to 342 HIV/AIDS trials are registered per year on ClinicalTrials.gov ^18^ and only three were registered for Middle East respiratory syndrome (MERS) coronavirus during 2007–2017 ^18^. While efforts being put into clinical trials were initially welcome, the vast majority of COVID-19 trials are at risk of being abandoned if they cannot recruit enough patients or if other trials on the same treatment provide conclusive results (favorable or unfavorable).

Thomas Chalmers highlighted in 1977 the need to ‘Randomize the first patient’ ^19^, and a reassuring 75.5% of trials are indeed randomized. However, we identified areas of concern. Most trials are not blinded, and even if placebos may be not available in such short time, blinding of outcome collection would be preferable. Blinding may not be required for mortality outcomes; however, mortality was rarely a primary outcome. Other objective outcomes commonly used according to the COVID-19 core outcome sets ^20^, such as hospitalization and mechanical ventilation, may still be impacted by subjective decisions and the awareness of the randomly allocated intervention, and thus may benefit from a blinded assessment. Half of the trials include fewer than 118 patients and many small trials were initiated after public registration of very large trials addressing similar questions. They may have some heterogeneity in design that might be desirable or focus on specific situations, for example early Phase 1 vaccine trials, but it seems unlikely that such small trials would add meaningfully to the overall evidence. The extensive worldwide discussions about limited evidence from small trials reflect the substantial uncertainty patients and decision-makers face about the merits of popular interventions, such as hydroxychloroquine ^21^.

For hydroxychloroquine, over 100 smaller studies with over 76,000 patients were planned in the first 100 days to investigate this single therapeutic option out of many potential options. This case, possibly fueled by media attention relayed by decision-makers and politicians, highlights the urgent need for early evidence-based research and priority setting. Such proliferation may reflect best intentions of clinical researchers to actively contribute to evidence generation and inform timely treatments locally instead of awaiting published evidence or using experimental treatments outside of clinical trials. It may also indicate a lack of research structures allowing them to contribute to larger, synergistic trials. With the emergence of results, the entire agenda may shift. Many hydroxychloroquine and chloroquine trials’ enrolment was temporally halted due to harmful effects in an observational study ^22^, the publication of which was subsequently retracted ^23^. However, the release of the randomized RECOVERY trial results showing no benefit (in fact, a trend for increased mortality) ^24^ with hydroxychloroquine and another “negative” trial on hydroxychloroquine-prophylaxis ^25^ created uncertainties about the feasibility (i.e. inability to recruit planned sample sizes) or futility (i.e. inability to demonstrate treatment effects) of all the ongoing and planned hydroxychloroquine trials.

There are excellent examples of how efficient structures allow for rapid response to evidence needs, such as the UK RECOVERY trial ^26^. Strongly endorsed and prioritized by authorities and medical representatives ^27^, it is running as a streamlined pragmatic platform trial in over 176 hospitals, randomizing over 12,000 patients in just over four months ^14^. It has already provided evidence on the lack of benefit for hydroxychloroquine ^24^ and lopinavir/ritonavir ^28^, and a reduction of mortality with dexamethasone ^29^ (still awaiting results for azithromycin, tocilizumab and convalescent plasma). Such key trials have had major impact on decision-makers such as the FDA revoking the Emergency Use Authorization of hydroxychloroquine ^30^ on June 15.

Conversely, we found other large trials with major recruitment difficulties. The DisCoVeRy trial, for example, was designed as an adaptive trial of 3200 patients, running in 35 countries. However, while DisCoVeRy recruited 758 patients in France, only one was recruited in the rest of Europe ^31^, as of June 17, 2020.

The lack of coordination in the research response created substantial research waste, exposed many patients to unnecessary risks, and harms medical progress by creating competition among trials investigating similarly promising therapeutic alternatives ^32– 34^. However, in absence of such desirable research synergies, all these scattered activities can and should be bundled to contribute to rapid evidence generation in living meta-analyses. The COVID-evidence database provides a unique opportunity to surveil the planned, ongoing and completed trials that can then be synthesized – it would only need systematic sharing of trial data.

As many countries are facing restrictions of movement and lifestyle at various severity levels, affecting the physical and mental health of billions of people, it is remarkable that not a single trial was initially planned to evaluate these measures. While the lack of controlled experiments evaluating their implementation may not be unexpected (given the initial urgency, ethical considerations, and organizational challenges), it would now be highly desirable that the de-implementation or re-implementation be subject to systematic evaluation in high-quality trials. The diverse options to ease or reinforce lockdown would be amenable to randomization, such as alternative time points or extents of re-opening schools or kindergartens, of ways to protect elderly in nursing homes, of home office programs, or of contact restrictions. Such evidence would be critical to inform future pandemics or the management of possible second waves of COVID-19, yet it was not on the initial agenda.

### Limitations

Several limitations merit attention. First, unclear reporting in registries might have introduced inaccurate results. Some ambiguously reported items required discussion among several reviewers but were resolved to the best of our ability. Second, we rarely identified protocols or manuscripts, precluding more detailed analyses of trial designs. Third, some control groups receiving “standard of care” interventions were not clearly described, likely some of these included interventions that were or are still under investigation in other trials. Fourth, we did not assess in details all the different outcomes being used and the blinding of the outcome collection was not systematically reported preventing us from fully apprehending the impact of the lack of blinding. Fifth, we may have missed a few cases of duplicate entries across registries or of multiple national parts of an international trial, thus slightly overestimating the number of trials but not affecting the overall interpretation. Sixth, we arbitrarily selected a period of the first 100 days, which is traditionally used to benchmark early outcomes of policies or presidencies. Finally, we do not assess the actual research output from all these early trials. This unprecedentedly fast-moving research body is scattered across data sources and registries without uniform updates. More definitive answers will require more time, but our results allow for the diagnosis of “system cracks” ^35^ that may become symptomatic in this pandemic, such as infrastructure limitations, and also identify best practices.

## Conclusion

The incredible volume and speed of trial research observed in the first 100 days of the COVID-19 pandemic should not hide the fact that in its early days the global clinical trial research agenda lacked clear coordination, efficiency and exploitation of synergies. There are excellent examples of very large trials implemented with impressive efficiency, likely providing the clearest evidence. However, early coordination and a unified approach are needed - otherwise futility and waste of resources may be prominent features of such an ambitious research agenda.

## Data availability

### Underlying data

All data underlying the results are available as part of the article and no additional source data are required.

The dataset for this study is provided on the Open Science Framework. It is based on continuously evolving data sources. With this second version, we corrected misclassifications/duplicates affecting 6 of the 689 previously included trials. Subsequent updates of COVID-evidence and the other sources may provide different datasets.

### Extended data

Open Science Framework: COVID-evidence: a living database of trials on interventions for COVID-19 / The worldwide clinical trial research response to the COVID-19 pandemic - the first 100 days; https://doi.org/10.17605/OSF.IO/GPMNA ^12^.

- 2020-10-13-Dataset_manuscript.xlsx. (Raw trial metadata.)
- 2020-06-03_COVe_Procedures_Variables_manuscript.pdf. (Procedures for screening and extracting data.)
- 2020-10-13-Extended_data_Manuscript.docx. (Extended data Figures 1–5 and Tables 1–5.)

Data are available under the terms of the Creative Commons Attribution 4.0 International license (CC-BY 4.0).
