# Supplementary material for: The worldwide clinical trial research response to the COVID-19 pandemic - the first 100 days
Source: F1000Res. 2020 Oct 26;9:1193. Originally published 2020 Oct 2. [Version 2] doi: 10.12688/f1000research.26707.2 (PMC7539080; doi:10.12688/f1000research.26707.2)
Supplement: Peer review history

# Peer review: Reviewer response for version 1

**DOI:** 10.5256/f1000research.29488.r72375

**Published:** 2020-10-06

**Authors:** Atle Fretheim

**Affiliations:** 1 Centre for Informed Health Choices, Norwegian Institute of Public Health, Oslo, Norway; 2 Faculty of Health Sciences, Oslo Metropolitan University, Oslo, Norway

The manuscript addresses an important topic, and was an interesting and relatively easy read. Thanks for that! 

 I have only managed to find one issue of some substance to comment on, and one very minor thing.

 In the Discussion section you raise the argument that lack of blinding is a problem, especially since many trials don’t have mortality as a main outcome. This is in line with the general understanding that “subjective” outcomes, i.e. outcomes based on some degree of judgement, are more prone to bias due to lack of blinding than “objective” outcomes (with regards to validity of outcome assessment) ^1^. However, I miss a mention of what outcomes the trials actually did include. All I find in the text about types of outcomes concerns whether or not mortality was included - nothing about what other types of outcomes were in use. This information is of some importance for assessing how crucial blinding is in these trials. I did manage to find the column on outcomes in the Extended Data file, but I think a sentence or two, or three, summarizing the general picture on outcome-types would be good to include in the main text.

 One minor details, on language, which I hesitate to mention being a non-native English speaker:

 Is there a word missing in this sentence (an “a” before “streamlined”, perhaps)? (Discussion, 5th paragraph):

 “Strongly endorsed and prioritized by authorities and medical representatives, it is running as streamlined pragmatic platform trial in over 176 hospitals, randomizing over 12,000 patients in just over four months”.

Is the work clearly and accurately presented and does it cite the current literature?

Yes

If applicable, is the statistical analysis and its interpretation appropriate?

Not applicable

Are all the source data underlying the results available to ensure full reproducibility?

Yes

Is the study design appropriate and is the work technically sound?

Yes

Are the conclusions drawn adequately supported by the results?

Yes

Are sufficient details of methods and analysis provided to allow replication by others?

Yes

Reviewer Expertise:

Research methods, health systems- and policy research, systematic review.

I confirm that I have read this submission and believe that I have an appropriate level of expertise to confirm that it is of an acceptable scientific standard.

## References

[1] : Cochrane Handbook for Systematic Reviews of Interventions (Version 6.1).2020; Reference source

# Response

**Published:** 2020-10-12

**Authors:** Perrine Janiaud

**Affiliations:** University Hospital Basel, Switzerland

Dear Atle Fretheim,

Thank you very much for your kind review and feedbacks. Some elements of answers to your comments below and we have submitted a revision:

- We have not assessed the outcomes in detail, mainly due to the heterogeneity of the reporting for the different outcomes. We unfortunately do not have additional data to present. However, we have added clarifications in the Discussion and Limits:

Discussion paragraph 3: "Blinding may not be required for mortality outcomes; however, mortality was rarely a primary outcome. Other objective outcomes commonly used according to the COVID-19 core outcome sets, such as hospitalization and mechanical ventilation, may still be impacted by subjective decisions and the awareness of the randomly allocated intervention and thus may benefit from a blinded assessment.”

Fourth limit: “Fourth, we did not assess in detail all the different outcomes being used, and the blinding of the outcome collection was not systematically reported preventing us from fully apprehending the impact of the lack of blinding”

- Thank you for spotting the missing “a”

# Peer review: Reviewer response for version 1

**DOI:** 10.5256/f1000research.29488.r72379

**Published:** 2020-10-05

**Authors:** Margaret McCartney

**Affiliations:** 1 General Practitioner and Freelance Writer, Glasgow, UK

I am very pleased to see this paper and congratulate the authors. It answers an important question on what research has been done and critically appraises them. This team already have my respect and admiration for their online COVID trials tracker. 

 These aren't criticisms: 

- I had difficulty trying to find what the small amount of trials using non drug/device interventions tested. I appreciated the appendixes with a list of all the trials, broken down into types of trials and interventions. It would perhaps help to draw out the non-drug interventions in full in the text especially as the authors list non-drug interventions that could be planned for testing. 
- In terms of further work, there should be data available about the trials that were planned in the next pandemic (some of this was in systems for epidemiological work across centres). I think we have missed a chance to plan non-drug intervention trials and appreciate the authors call to do this. It might be helpful to know how many planned trials actually happened to help model non drug trial options. 
- Is there any way to graphically explain types of funders in relation to types of trial? It might be a way to hold funders to account - I suspect this will show that it is better to fund fewer bigger trials but I don't know - it might be helpful for funders reading this to appreciate where to put their dollar. 

Thank you for writing this, I think it does a great job of holding the research communities to account.

Is the work clearly and accurately presented and does it cite the current literature?

Yes

If applicable, is the statistical analysis and its interpretation appropriate?

I cannot comment. A qualified statistician is required.

Are all the source data underlying the results available to ensure full reproducibility?

Yes

Is the study design appropriate and is the work technically sound?

Yes

Are the conclusions drawn adequately supported by the results?

Yes

Are sufficient details of methods and analysis provided to allow replication by others?

Yes

Reviewer Expertise:

I am a general practitioner with an interest in evidence based medicine.

I confirm that I have read this submission and believe that I have an appropriate level of expertise to confirm that it is of an acceptable scientific standard.

# Response

**Published:** 2020-10-12

**Authors:** Perrine Janiaud

**Affiliations:** University Hospital Basel, Switzerland

Dear Margaret McCartney,

Thank you very much for your kind review and feedbacks. Some elements of answers to your comments below and we have submitted a revision:

- We have added a few examples in the Results section “Intervention to treat COVID 19 […] Regarding non-drug interventions, 28 investigated procedures (e.g. renal replacement therapy), 8 devices (e.g. various respiratory devices), and 27 trials investigated other non-drug interventions (e.g. physical activity and pulmonary rehabilitation).”
- We agree, there’s clearly a need to learn from the challenges and successes of the COVID-19 research agenda. Such knowledge would help for future health challenges but on a more immediate level it could definitely inform what would be the best design for non-drug trials. Even more so important, as many countries are currently taking actions to reinforce restrictions.
- As the funding type was unclear for 41.8% of the assessed trials (i.e. a public/not-for-profit sponsor was reported but the absence of an industrial sponsor does not exclude an industrial funder), we believe that a graph may not be useful to reflect the reality.
